# Intra-tumoral microbial community profiling and associated metabolites alterations of TNBC

**DOI:** 10.3389/fonc.2023.1143163

**Published:** 2023-10-12

**Authors:** Yi Wang, Dingding Qu, Yali Zhang, Yiping Jin, Yu Feng, He Zhang, Qingxin Xia

**Affiliations:** ^1^ Department of Pathology, Affiliated Cancer Hospital of Zhengzhou University, Zhengzhou, China; ^2^ Henan Medical Key Laboratory of Tumor Pathology and Artificial Intelligence Diagnosis, Zhengzhou, China; ^3^ Zhengzhou Key Laboratory of Accurate Pathological Diagnosis of Intractable Tumors, Zhengzhou, China

**Keywords:** TNBC, triple-negative breast cancer, FFPE, formalin-fixed paraffin-embedded, microbiota, metabolome, tumor-infiltrating lymphocytes

## Abstract

Triple-negative breast cancer (TNBC) presents significant challenges to female health owing to the lack of therapeutic targets and its poor prognosis. In recent years, in the field of molecular pathology, there has been a growing focus on the role of intra-tumoral microbial communities and metabolic alterations in tumor cells. However, the precise mechanism through which microbiota and their metabolites influence TNBC remains unclear and warrants further investigation. In this study, we analyzed the microbial community composition in various subtypes of breast cancer through 16S rRNA MiSeq sequencing of formalin-fixed, paraffin-embedded (FFPE) tissue samples. Notably, *Turicibacter*, a microbe associated with cancer response, exhibited a significantly higher abundance in TNBC. Similarly, mass spectrometry-based metabolomic analysis revealed substantial differences in specific metabolites, such as nutriacholic, pregnanetriol, and cortol. Furthermore, we observed significant correlations between the intra-tumoral microbiome, clinicopathological characteristics, and human epidermal growth factor receptor-2 expression(HER2). Three microbial taxa (*Cytophagaceae, Conexibacteraceae*, and *Flavobacteriaceae*) were associated with tumor-infiltrating lymphocytes(TILs), which are indicative of antitumor immunity. This study creatively utilized FFPE tissue samples to assess intra-tumoral microbial communities and their related metabolic correlations, presenting avenues for the identification of novel diagnostic biomarkers, the development of therapeutic strategies, and the early clinical diagnosis of TNBC.

## Introduction

Breast cancer (BC) is the most prevalent malignant tumor among females, and its incidence and mortality rates are on the rise, including that of triple-negative BC (TNBC) (1). TNBC is characterized by the absence of the estrogen receptor (ER), progesterone receptor (PR), and human epidermal growth factor receptor-2 (HER2). Compared to non-TNBC, TNBC is the most aggressive subtype of BC and currently faces limited treatment options (2, 3). Owing to the differences in clinical manifestations between TNBC and non-TNBC, exploring the correlated mechanisms for developing novel therapeutic strategies and improving the prognosis of patients with TNBC is imperative.

Cancer progression is influenced by changes in various components of the tumor microenvironment (TME). Alterations in stromal composition, including the surrounding immune cells, lymphocytes, blood vessels, extracellular matrix, fibroblasts, and certain signaling molecules, can impact host metabolism, immune responses, and cancer-driving molecular alterations, thereby influencing tumor development and response to cancer therapy (4). Recent research has highlighted the complexity and significance of the relationship between the microbiome and cancer. Certain microbial species within tumors, as a host factor, can stimulate an inflammatory state or immune response, thereby promoting carcinogenesis and tumor progression (5). Furthermore, the diversity and composition of the bacterial community have been associated with different histological classifications of cancer, reflecting the distinct TME characteristics (6, 7). Consequently, the tumor microbiota may not only serve as a diagnostic tool for better cancer classification but also influence tumor behavior and patient prognosis based on the properties of the microbes themselves (8). Emerging evidence suggests a potential link between the microbiota and carcinogenic metabolites that contribute to tumor progression (9). As “tumor foragers,” the tumor microbiota plays a crucial role in regulating the host metabolome by establishing a biological “digester” (10). Cancer metabolism can modulate the TME to facilitate cancer progression through the release of amino acids, nucleotides, organic acids, and lipids that fulfill the metabolic demands of the body.

Studies have provided evidence of a significant association between microbial composition and the development of BC. Urbaniak et al. demonstrated that patients with BC exhibited a relatively high abundance of *Lactobacillus, Hydrogenophaga*, and *Fusobacterium* compared to those with benign breast lesions or normal tissue (11). More importantly, the gastrointestinal microbiota plays a crucial role in regulating estrogen levels, and estrogen, in turn, influences BC development through host–microbe interactions (12). To investigate this further, we first analyzed the differences in microbial composition between non-TNBC and TNBC through 16S rRNA sequencing of formalin-fixed, paraffin-embedded (FFPE) BC tissue samples. Additionally, to explore the interactions between microbes and metabolites, we examined metabolite abundance in FFPE BC tissue samples through liquid chromatography–mass spectrometry (LC–MS) analysis, seeking to identify relevant metabolic signaling pathways that may potentially be involved in BC molecular mechanisms. Our findings may aid in the discovery of novel biomarkers in TNBC and pave the way for the development of effective therapeutic strategies.

## Materials and methods

### Human BC tissue samples

Surgical specimens of BC were collected from the Zhengzhou University Affiliated Cancer Hospital (Zhengzhou, China) between 2014 and 2016. Overall the clinical variables between TNBC group and non-TNBC group were comparable, with no significant difference in the age, BMI, tumor size and parity (**Supplementary Table 1**). FFPE tissue samples were sliced into 5-mm-thick sections and stained with hematoxylin and eosin (HE). Slices were evaluated by two or more pathologists. The study was approved by the ethics committee, and all patients provided informed consent. The patients from whom the samples were obtained did not undergo standard BC therapy, i.e., chemotherapy and/or radiotherapy.

### Immunohistochemistry and tumor-infiltrating lymphocytes (TILs) evaluation

We assessed the protein expression levels of ER, PR, and HER2 in the BC tissue samples. The tissue sections were stained using the Ventana BenchMark ULTRA automatic immunohistochemical staining platform (Ventana Medical Systems Inc., Tucson, AZ, USA) and observed under a microscope (Olympus BX41). Rabbit monoclonal primary antibodies against ER (SP1 Roche), PR (1E2 Roche), HER2/NEU (Clone 4B5 Roche), and PD-L1 (SP142 Roche) were used, and the OptiView DAB immunohistochemistry Detection Kit and OptiView Amplification Kit (Ventana Medical Systems Inc.) were used for subsequent analysis. HER2 staining was scored according to the HER2 Testing Guidelines for Breast Cancer (2019 edition) (13). ER- and PR-positive staining was defined according to the ASCO/CAP guidelines (14).

In the HE slides, TILs were defined as a continuous parameter by two experienced pathologists. TILs on the boundaries of the cancer were included, while those in the tumor bed were excluded, and they were scored based on the area occupied over the entire region. The final percentage of TILs was calculated as the average of the specimens and was not restricted to hotspots. All evaluations were performed according to the criteria recommended by the International TILs Working Group (2014) (15). TILs were assessed as a continuous parameter, and reported scores were rounded up to the nearest 10%.

### DNA extraction and high-throughput 16S rRNA gene sequencing

We performed 16S rRNA sequencing on 22 BC samples, comprising 13 TNBC and 9 non-TNBC samples. For microbiota analysis, 10-µm thick sections were used. Total DNA was extracted using the QIAamp DNA FFPE Tissue Kit (QIAGEN, Redwood City, CA, USA) according to the manufacturer’s protocol. The quality of the extracted DNA was assessed by subjecting each sample to 1% agarose gel electrophoresis at room temperature. DNA concentration and purity were determined using a NanoDrop 2000 spectrophotometer. To amplify the bacterial 16S rRNA gene V3–V4 region, we used the following primers: 338F: 5′-ACTCCTACGGGAGGCAGCAG-3′ and 806R: 5′-GGACTACHVGGGTWTCTAAT-3′. The mixed PCR products were purified and quantified using a Quantus™ fluorometer (Promega). Subsequently, we constructed a database using the NEXTFLEX Rapid DNA-SEQ Kit and performed sequencing with 2 × 250 bp chemistry on the Illumina MiSeq PE300 platform (Illumina, San Diego, CA, USA) (16, 17).

### Sequencing data analysis

The obtained gene sequences were attached to unique bar codes and clustered into operational taxonomic units (OTUs) with 97% identity, utilizing the USEARCH software (version 7.0, http://drive5.com/uparse/). Each sequence was then compared with the Silva database (SSU132) using the RDP classifier (http://rdp.cme.msu.edu/), and the comparison threshold was set to 70% to obtain the annotation results for species classification. To evaluate species richness based on OTU values, we performed dilution curve analysis (17). Principal coordinate analysis (PCoA) was conducted using the R package (http://www.r-project.org/) to assess the differences in microbiota between the groups (18). For exploring the extent to which certain the microbiota, distance-based redundancy analysis was performed at the OTU level, employing Bray–Curtis distances. Bacterial abundance and diversity were compared using an independent t-test. To evaluate differentially abundant taxa, we used linear discriminant analysis coupled with effect size (LEfSe) (19).

### Metabolite extraction and LC–MS untargeted metabolomic analysis

Metabolites from the 22 FFPE samples were extracted by preparing 20-µm thick tissue sections. Details regarding sample preparation, LC–MS analysis, data quality management, and compound identification can be found in the Supplementary Information (20). For LC–MS analysis, we employed an ultra-high-performance liquid chromatography-triple time-of-flight mass spectrometry system (AB SCIEX LLC). The LC–MS data were then imported into the metabolomics processing software Progenesis QI (Waters Corporation, Milford, MA, USA).

### Statistical analyses

Statistical analysis was conducted using SPSS software (version 22.0; SPSS Inc., Chicago, IL, USA). Clinical characteristics were assessed using the χ^2^ test, while t-tests were utilized to determine differences between two groups. Pearson’s correlation was used to analyze the correlation between microbial species at the phylum level and relevant environmental factors or metabolites, with the numerical matrix visually displayed using heatmaps. The color depth in the heatmap corresponds to the size of the data. Statistical significance was considered at P < 0.05.

## Results

### Abundance and diversity of microbiota in FFPE BC tissue samples

**Figure 1:**
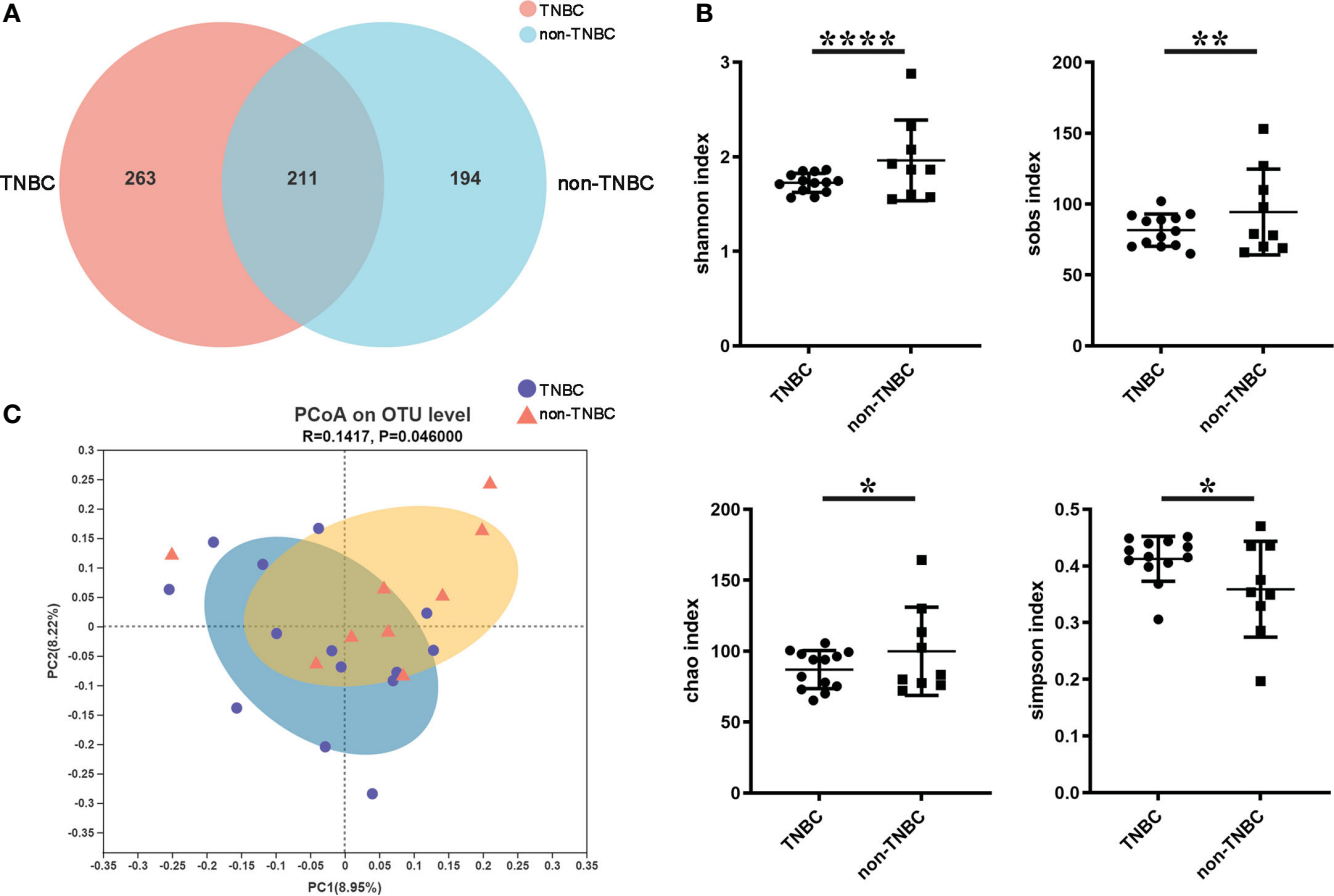
Tumor microbial diversity in TNBC and non-TNBC by FFPE. **(A)** A Venn diagram displaying the overlaps and unique OTUs between TNBC and non-TNBC groups. 263 of 474 OTUs were unique in TNBC. **(B)** Shannon index, Sobs index, Chao index, Simpson index estimated microbial diversity in the two groups. **(C)** Beta diversity was calculated based on unweighted unifrac by PCOA.

To investigate the microbial abundance and diversity in BC, all paraffin-embedded tissue samples were subjected to 16S rRNA sequencing. To eliminate any potential contaminants, simultaneous sample detection was performed after quality filtering. The rarefaction curves of all the samples (**Figures S1A, B**) validated the adequacy of the sampling efforts. A Venn diagram revealed 668 OTUs, including 211 overlapping OTUs (**Figure 1A**). Alpha diversity, based on Shannon, Sob, Simpson, and Chao indices, was significantly lower in patients with TNBC than in the other groups (P < 0.0001, 0.0032, 0.0190, and 0.0102, respectively) (**Figure 1B**). Beta-diversity was calculated using unweighted UniFrac at the OTU level, and PCoA showed that tumor microbial communities varied among the samples (P = 0.046) (**Figure 1C**). These results suggest that the diversity of the tumor microbiota varies across BC subtypes.

### Alterations in tumor microbiota composition are associated with TNBC

**Figure 2:**
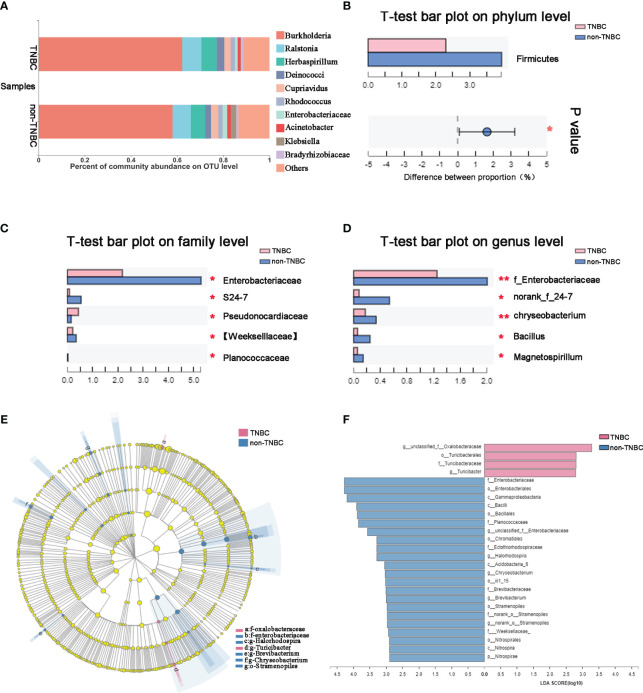
Tumor microbial communities are different between TNBC and non-TNBC. **(A)** Composition of microbiota at the OTU level between TNBC and non-TNBC. Significantly altered tumor microbiota between the TNBC and non-TNBC groups at **(B)** phylum, **(C)** family and **(D)** genus levels is represented by the Wilcoxon rank-sum test. f, family. *P<0.05; **P<0.01. **(E)** The specific characterization of tumor microbiota was analyzed by linear discriminant analysis (LDA) effect size (LEfSe) method (http://huttenhower.sph.harvard.edu/lefse/) between TNBC and non-TNBC. Each node represents a specific taxonomic type. Yellow nodes show there is no difference between non-TNBC and TNBC; red nodes show the taxonomic types with more abundance in TNBC group, while blue nodes represent the taxonomic features with more abundance in non-TNBC group. **(F)** LDA score computed from features differentially abundant between TNBC and non-TNBC. The criteria for feature selection is log LDA score > 2.

To identify the composition of the intra-tumoral microbial community in each sample, we compared phylotypes with an abundance greater than 0.01% of the total OTU. *Proteobacteria* was the predominant phylum, accounting for 88.4% and 87.2% in the two groups. *Actinobacteria* (3.6% and 3.3%), *Firmicutes* (2.5% and 3.9%), and *Bacteroidetes* (0.97% and 1.67%) were enriched at the phylum level (**Figure S1C**). The abundance at the genus and OTU levels in the TNBC group differed from that in the non-TNBC group (**Figure 2A**; **Figure S1D**). Student’s *t*-test was performed to analyze the differences in the microbial communities between the two groups. The abundance of *Firmicutes, Enterobacteriaceae*, and *Weekselllaceae* was lower in the TNBC group than in the non-TNBC group (P = 0.039, 0.004, and 0.047, respectively), whereas the abundance of *Pseudonocardiaceae* was significantly increased (P = 0.005) (**Figures 2B, C**). **Figure 2D** shows similar results. LEfSe analysis revealed enriched microbial abundance in the two groups (**Figures 2E, F**). *Turicibacter* was significantly more abundant in the TNBC group than in the other group and may be a key factor associated with inflammatory and cancer responses in patients with TNBC. Therefore, the diversity and richness in the TNBC group appear to be much lower compared to those in the non-TNBC group.

### Composition of detected metabolites in FFPE tissue samples of the TNBC and non-TNBC groups

**Figure 3:**
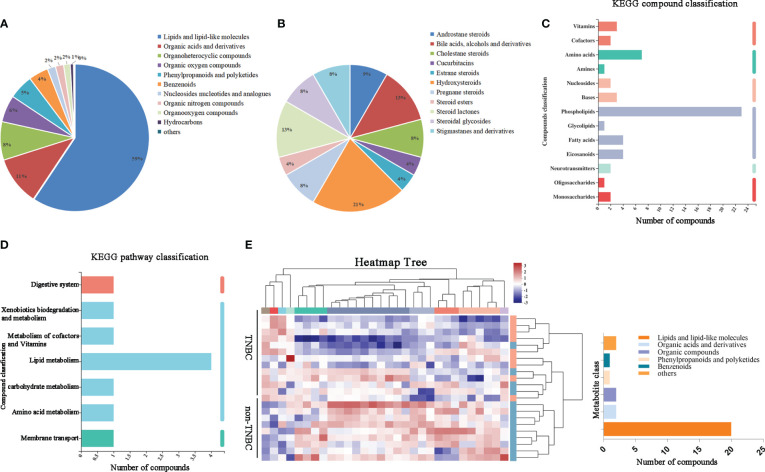
Identified tumor metabolites composition and diversity in BC by FFPE. **(A)** Pie chart based on counts of HMDB chemical taxonomy for different classes of metabolites in all breast cancer samples. **(B)** Pie chart based on counts of HMDB chemical taxonomy in Steroids and Steroid derivative content. **(C)** KEGG compound classification and **(D)** KEGG pathway classification: metabolites detected and annotated in breast cancer FFPE tissues. **(E)** Hierarchical clustering analysis for identification of different metabolites by comparison of the TNBC and non-TNBC group. Each column in the figure represents a sample, the expression level of the samples is indicated as a colored band on top of the heat map.

We hypothesized that metabolites may be affected by the microbiota in tumors. To investigate this, we first assessed the component superclasses of the metabolites and determined their distribution in FFPE samples (**Figure 3A**). **Figure 3B** shows the proportions of 24 different steroids and steroid derivatives in the component classes. By comparison with the Kyoto Encyclopedia of Genes and Genomes (KEGG) database, we classified genes according to their functions (**Figure 3C, D**). Subsequently, we visualized the metabolite abundance between the two groups using a heatmap and found that both groups had similar metabolic abundances. Among these metabolites, steroids and steroid derivatives exhibited relative richness, suggesting that they might exert a notable influence on BC (**Figure 3E**).

### Differentially abundant metabolites between TNBC and non-TNBC FFPE tissue samples

**Figure 4:**
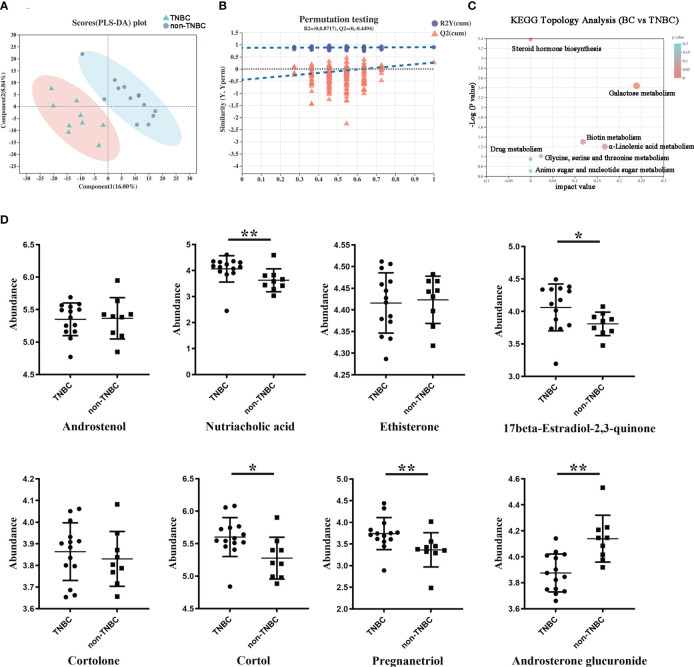
Differentially abundant metabolites between TNBC and non-TNBC FFPE samples. **(A)** PLS-DA analysis displaying the two group’s classification comparison by the first two PCs. **(B)** PLS-DA model evaluation by permutation test. **(C)** EGG topology analysis shows that metabolites in the TNBC and non-TNBC groups have differentially accumulated [impact value on X-axis] and have significantly changed [-log10(p) on Y-axis]. Bubble size represents impact value; the bigger the bubble, the more important the pathway. **(D)** The metabolites difference from different subclass between the TNBC and non-TNBC groups. (*P < 0.05; **P < 0.01).

Next, we compared the metabolite abundance between the FFPE tissue samples of the TNBC and non-TNBC groups through LC–MS analysis. The differential abundance between the two groups was determined using a permutation t-test (**Figure 4A**). PLS-DA revealed differences between the two groups based on the first two principal components (PC1: 16%; PC2: 8.84%) (**Figure 4B**). Compared to the non-TNBC group, the TNBC group exhibited a lower abundance of metabolites. As shown in **Figure S3**, several specific metabolites differed between the TNBC and non-TNBC groups, and variables with higher VIP scores were considered important for classification.

To gain a comprehensive understanding of the detected metabolites in the FFPE tissue samples, we performed metabolite categorization (superclass, class, subclass, and metabolic pathway) based on the Human Metabolome Database (http://www.hmdb.ca/) and KEGG (http://www.genome.jp/kegg). Affected metabolic pathways were identified by KEGG topology analysis (**Figure 4C**). Enrichment analysis revealed that these metabolic pathways may influence the biological behavior of BC. Given the loss of hormone expression in the TNBC group, we specifically investigated the metabolite profiles corresponding to the steroid and steroid derivative classes between the two groups. Nutriacholic acid, 17beta-estradiol -2,3-quinone, and pregnanediol were found to be more abundant in the TNBC group than in the non-TNBC group, whereas cortol and androsterone glucuronide were more abundant in the non-TNBC group (**Figure 4D**). However, androstenol, ethisterone, and cortolone levels were not significantly different between the two groups. These metabolites exhibiting statistically different abundances may serve as robust markers and contribute to our understanding of the biological characteristics of patients with TNBC. Overall, these results strongly suggest a TNBC group-specific metabolomic abundance, signatures, and metabolic differences.

### Correlations between tumor microbiota and differentially abundant metabolites in BC

**Figure 5:**
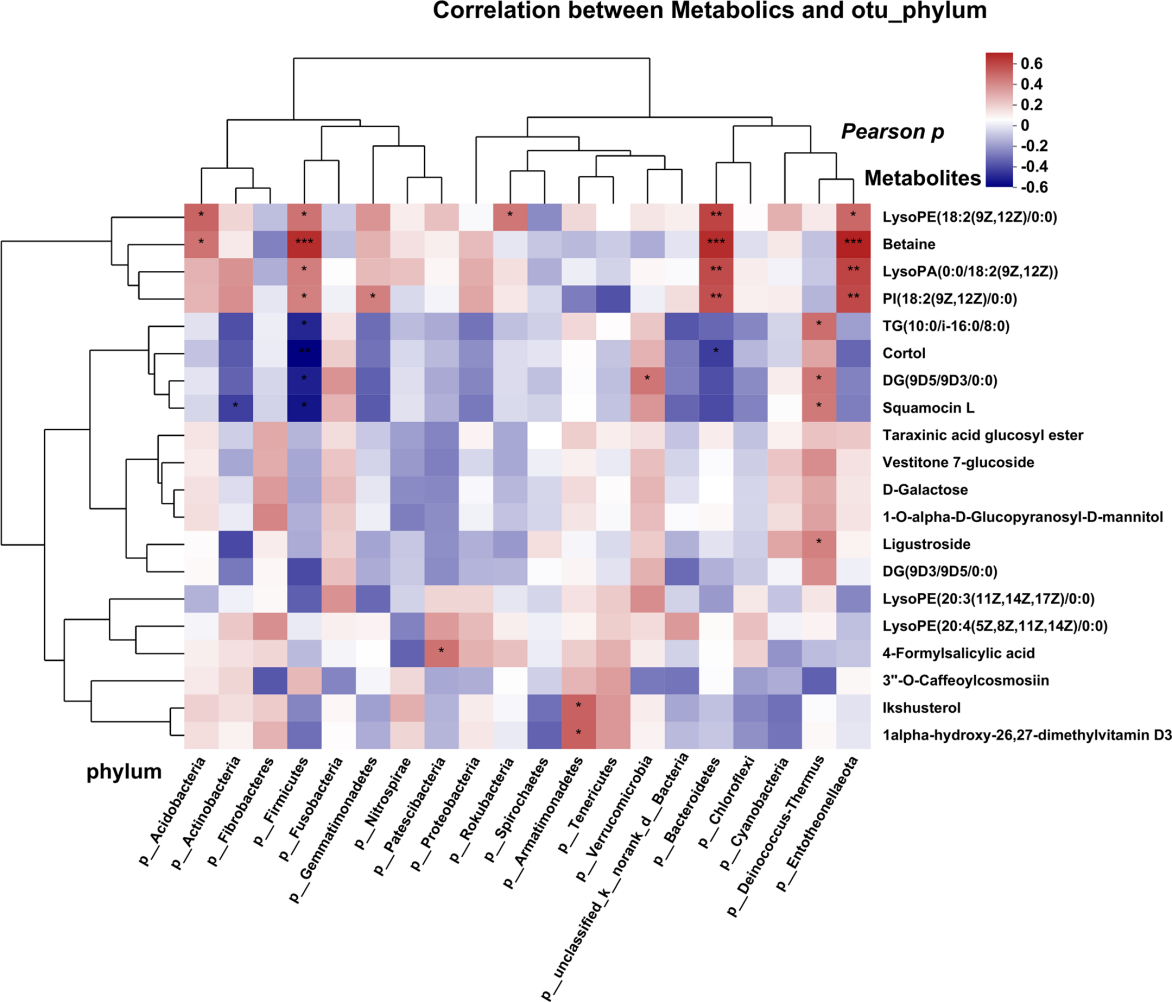
Correlation analysis of microbes and metabolites, each lattice represents a coefficient by Pearson’s correlation analysis, each row represents a phylum, each column represents a metabolite. Red represents a positive correlation and blue represents a negative correlation. (*P < 0.05; **P < 0.01, ***P<0.001).

To investigate the potential relationship between tumor microbiota and metabolites, we examined the correlations between several bacteria at the phylum level and certain metabolites (**Figure 5A**). The results revealed a positive correlation between several microbes (*Acidobacteria* and *Firmicutes*) and the levels of betaine. Conversely, we observed a negative correlation between several microbes (*Firmicutes* and *Bacteroidetes*) and the abundance of the metabolite cortol in the FFPE tissue samples. Similar results were obtained for several genera at the family and genus levels, which exhibited correlations with certain metabolites as determined by Pearson analysis (**Figures S4A, B**). Lipids and lipid-like molecules of metabolic species were more closely associated with microorganisms, implying that they may potentially influence molecular mechanisms and related pathways in BC.

### Correlation between clinical indices and the BC microbiome

**Figure 6:**
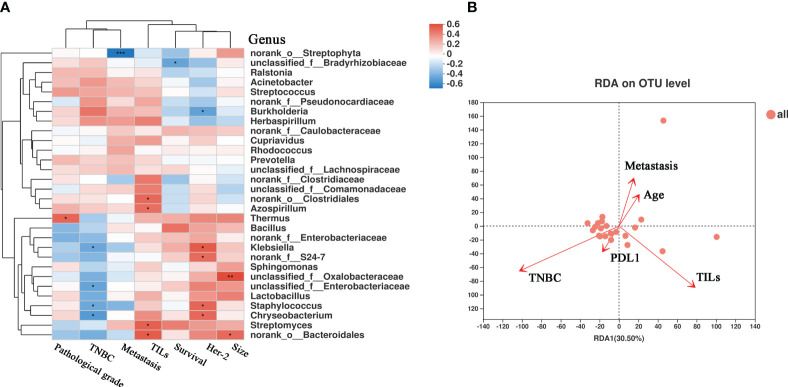
The correlation heatmap between clinical relative indices and the microbiome in breast cancer. **(A)** Clinical relative indices include pathological grade, TNBC or not, distant metastasis states, survival, HER-2 state and size. Red represents a positive correlation and blue represents a negative correlation. **(B)** Distance-based Redundancy Analysis(db-RDA) plot showing the relationship of age, metastasis, PD-L1 expression, TILs, and TNBC to the microbial community structure. (*P < 0.05; **P < 0.01).

We also investigated the correlations between the microbiota and various clinical parameters, including pathological grade, tumor size, metastasis, lymphatic metastasis, survival, TNBC status, TILs, and the expression of HER2. These clinical parameters were correlated with the bacterial genus, as shown in the heatmap in **Figure 6A**. Notably, *Thermus* showed a positive correlation with pathological grade (r = 0.464, P = 0.029). Moreover, we observed a positive correlation between HER2 expression and *Klebsiella* (r = 0.500, P = 0.017) and *Staphylococcus* (r = 0.462, P = 0.030), and a negative correlation was observed between HER2 expression and *Burkholderia* (r = -0.469, P = 0.027). We also analyzed correlations between TILs and microbes. TILs were positively correlated with *Clostridiales* (r = 0.452, P = 0.034), *Bacteroidales* (r = 0.496, P = 0.018), and *Azospirillum* (r = 0.431, P = 0.044), but negatively correlated with *Streptophyta* (r = -0.161, P = 0.024). The correlation between PD-L1 expression, lymph node metastasis, TILs, distant metastasis, and microbes (**Figures S1E–H**) further emphasized the notable role of tumor microbes in patients with BC. As shown in **Figure 6B**, RDA at the OTU level revealed a relationship between the intra-tumoral microbial community and certain clinical indices. TILs were positively correlated with bacteria such as *Cytophagaceae*, *Conexibacteraceae*, and *Flavobacteriaceae* (**Figures S5A–C**). PCoA did not reveal significant differences in bacterial communities, short-term survival, long-term survival, TIL status, or lymph node metastasis between the groups (**Figures S2C, E, G**). Tumor microbial characteristics were analyzed through LEfSe, which revealed marked differences in the predominance of bacterial communities (**Figures S2D, F, H**). The samples were categorized into four groups based on the presence or absence of TNBC and TILs. The beta diversity among the groups displayed significant differences (P = 0.0002) (**Figure S2A**), and the abundance of the bacterial communities also showed marked differences (**Figure S2B**). These results further highlight the importance of microbial abundance and diversity between TNBC and non-TNBC, with TILs potentially serving as a prognostic indicator of microbial diversity in TNBC. To some extent, these results validate the critical communication between microbiota, metabolites in the TME, and clinical factors.

## Discussion

Recent studies have underscored the critical importance of the relationship between tumor microbiota and the TME in understanding tumor development. BC tissues exhibit distinct microbiomes with an enrichment of specific species. In particular, TNBC demonstrates a unique microbial microenvironment that profoundly influences the biological behavior of tumors. Despite advancements in understanding the underlying tumor biology of TNBC, clinical outcomes for patients with TNBC unfortunately remain poor (21). Therefore, the establishment of a novel microbial typing system for TNBC is essential to providing valuable insights and assistance for patients with TNBC. Our study is the first to investigate the impact of intra-tumoral microbiota on TNBC using FFPE tissue samples and to validate the differences in microbiota between TNBC and non-TNBC. In our previous study, we explored the potential relationship between hepatocellular carcinoma and tumor microorganisms using paraffin-embedded tissue specimens and found that *Pseudomonas* is a differentially abundant microbe between cancer and adjacent tissues, thereby presenting potential avenues for the early clinical diagnosis and treatment of liver cancer (6). In this study, we analyzed a total of 22 BC FFPE tissue specimens through 16S rRNA MiSeq sequencing. The results showed that TNBC exhibited lower abundance of various microbes and that the microbiome diversity displayed significant differences in alpha and beta diversity owing to tumor heterogeneity and distinct molecular subtypes of BC. Notably, specific bacteria, such as *Firmicutes* and *Enterobacteriaceae*, were significantly decreased in TNBC, and the microbial diversities were significantly different between TNBC and non-TNBC. Firmicutes, an adipocyte-derived bacteria, can directly or indirectly impact BC tissue through toxin or enzyme production and is associated with bacterial load and immune cell infiltration in BC (22, 23). Jeongshin et al. showed that the abundance of Firmicutes can influence diseases related to obesity in patients with BC and is associated with a poor prognosis, which aligns with our findings (24). Firmicutes thus hold potential as a diagnostic marker and risk factor for BC. Similarly, the family Enterobacteriaceae, which includes *Escherichia coli*, exhibited a relatively lower abundance in patients with TNBC, indicating decreased involvement of signaling pathways in the TNBC microenvironment (10). The differences in microbial communities among different BC subtypes suggest that microbial markers may serve as noninvasive diagnostic tools and guide the development of therapeutic strategies for patients with BC.

The microbiota can influence the regulation of various metabolic pathways associated with energy homeostasis, nutritional intake, and immune balance (22). Metabolites produced by these bacterial species can profoundly impact molecular events in BC (25). Recent studies have elucidated unique and common viruses, bacteria, and fungi, and various metabolic pathways exhibit distinct patterns in each type of BC (26). Wang et al. demonstrated that the crosstalk between microbiota, metabolites, and the immune system could serve as a novel therapeutic strategy for TNBC (27). They discovered a new metabolite, trimethylamine N-oxide, which affects the treatment of TNBC. Additionally, 17beta-estradiol-2,3-quinone, a reactive metabolite of estrogen, is considered responsible for estrogen-induced genotoxicity and serves as a significant predictor of BC. 17beta-estradiol-2,3-quinone can convert to estrogen catechols and undergo oxidation to form quinones. Accumulation of estrogen quinones along with the DNA damage contribute to estrogen-induced carcinogenesis. In our study, we found that 17beta-estradiol-2,3-quinone was more abundant in the TNBC group, suggesting that the disparity in estrogen disposition and the subsequent elevation of the cumulative quantity of 17beta-estradiol-2,3-quinone in the body may play a role in the development of BC (28, 29). Furthermore, lipid derivatives associated with microbes may effectively reduce BC risk. When changes occur in lipid pathways, it can affect the availability of structural lipids for membrane synthesis, lipid synthesis, and degradation that contribute to energy homeostasis and cell signaling functions (30). We observed a relationship between tumor microbiota and metabolites, with *Bacillus* showing a significant difference between the TNBC and non-TNBC groups and being associated with betaine metabolites. Regrettably, no other microorganism with differential abundance was found to be associated with metabolites. Moreover, the relationship between tumor microbiota and metabolites in different subtypes of BC was not adequately compared. These results suggest that a deeper understanding of the correlation between microbes and their metabolites in BC samples may offer valuable insights for the development of diagnostic, therapeutic, and preventive strategies.

In line with our results, TNBC exhibits unique clinicopathological features that can influence therapeutic decisions (**Supplementary Table 2**). Pathological grade and TILs exhibited statistically significant differences in the TNBC group (31). Additionally, we elucidated an association between clinicopathological factors and the BC microbiota. The results showed that certain microbes and metabolites were significantly correlated with survival, lymphatic metastasis, distant metastasis, TNM stage, pathology grade, HER2 expression, ER status, and PR status. For instance, *Klebsiella, Staphylococcus, Burkholderia*, and *Thermus* were found to be correlated with HER2 and pathological grade. Meng et al. also highlighted significant differences in tumor microbiota and metabolism among patients with BC with different histological grades (23). Importantly, amino acids and fatty acids displayed the most pronounced differences between TNBC and non-TNBC, consistent with the results of other studies (32). TILs provide novel insights into the crosstalk between microbiota and metabolites, which may potentially influence therapeutic strategies and benefit patients with TNBC. TILs play a crucial role in the response to immune checkpoint inhibitor therapy by increasing PD-L1 expression and are closely associated with the prognosis of BC, especially TNBC (33). The microbiota can regulate estrogen metabolism and tumor immune activation. TILs were significantly different between the TNBC and non-TNBC groups. This study is the first to elucidate that *Clostridiales, Bacteroidales, Azospirillum*, and *Streptophyta* are correlated with TILs. The association between TILs and PD-L1 could have implications for prognostics and may lead to the discovery of new targets for chemotherapy in BC (34, 35). Overall, our findings indicate an innate relationship among the ecological environment, immunity, and treatment efficiency in patients with BC. Additionally, besides metabolism, the microbiota could be utilized to monitor the efficiency of chemotherapy or chemotherapy resistance (36).

In conclusion, through FFPE tissue samples, our study highlights the antitumor role of intra-tumoral microbiota in TNBC and indicates a correlation between bacterial biomass in the tumor and clinical factors. Additionally, our results indicate that tumor microbiota possibly modulates the immune microenvironment and elicits an antitumor response through TILs. Furthermore, our study provides novel insights into the interplay between the microbiome and metabolome, which may pave the way for the discovery of clinical diagnostic indices for BC. Nevertheless, this study has several limitations. First, the sample size was relatively small, which might have hindered the detection of a potential relationship between the microbiome and the prognosis of patients with BC. While several positive results were obtained, a larger sample size will be necessary in future research to validate our findings. Second, considering the detailed study of differences across BC subtypes, another limitation of the study is the absence of a control group comprising adjacent normal breast tissues for a comparison of the microbial and metabolic differences between benign and cancerous breast tissues, which could corroborate the results of this study. In addition, this study primarily focused on the direct influence of microbial metabolites on BC tissues to modulate TME. However, validating whether microbial metabolites affect tumor cell function requires cytological and molecular mechanistic experiments. Despite these limitations, we propose that biomarkers targeted at tumor microbiota or metabolites could hold promise as diagnostic and therapeutic tools for TNBC.

## Data availability statement

The datasets presented in this study can be found in online repositories. The names of the repository/repositories and accession number(s) can be found in the article/**Supplementary Material**.

## Ethics statement

The studies involving humans were approved by Cancer Hospital of Zhengzhou University of the ethics committee. The studies were conducted in accordance with the local legislation and institutional requirements. The human samples used in this study were acquired from Surgical samples of inpatients in our hospital. Written informed consent for participation was not required from the participants or the participants’ legal guardians/next of kin in accordance with the national legislation and institutional requirements. The studies were conducted in accordance with the local legislation and institutional requirements.

## Author contributions

This work was designed by YW, HZ and QDD. ZH and DQ provided essential reagents and materials. YJ collected clinical samples. DQ and HZ conducted laboratory assays. YZ performed the statistical analyses and interpreted data. YW drafted the manuscript. QX and HZ revised the study. YZ and YF performed the statistical analyses and interpreted data. All authors contributed to the article and approved the submitted version.

## Supplementary material

Supplementary Figure 1 Composition and differences of tumor microbiota in different clinical indices.

Supplementary Figure 2 The diversity and abundance of microbiota in other related elements.

Supplementary Figure 3 Variable importance in projection (VIP) scores of tumor metabolites in TNBC and non-TNBC groups.

Supplementary Figure 4 Correlation analysis of microbes and metabolites.

Supplementary Figure 5 The correlation between the intra-tumoral microbiota and clinicopathological characteristics.
