# Supplementary material for: Intra-tumoral microbial community profiling and associated metabolites alterations of TNBC
Source: Front Oncol. 2023 Oct 12;13:1143163. doi: 10.3389/fonc.2023.1143163 (PMC10602718; doi:10.3389/fonc.2023.1143163)
Supplement: Supplementary file 1 [file DataSheet_1.doc]

# Supplementary Methods

**Metabolite extraction**

The metabolites from 22 FFPE samples were extracted by sectioning 20 µm. Samples were placed in a 1.5 mL centrifuge tube, and 400 µL of the extract was added (acetonitrile:methanol = 1:1). After de-paraffinization procedure, mixing for 30 s, and low-temperature ultrasonic extraction for 30 min (5 ℃, 40 kHz), the sample was placed on ice for 15 min, centrifuged at 14,000 *g* for 15 min, and then the supernatant was transferred into a new micro-centrifuge tube and chilled on ice for 10 min. Next, the 120 µL complex solution (acetonitrile:water = 1:1) was added to redissolve the sample, followed by low-temperature ultrasonic extraction for 5 min (5 ℃, 40 kHz) and centrifugation at 13,000 *g* for 5 min at 4 ℃. The supernatant was stored at −80 °C until metabolic analyses.

For Quality assurance (QA)/QC purposes, a number of additional samples were included and a selection of QC compounds was added to every sample. These QC samples were primarily used to evaluate the process control for each study as well as aiding in the data curation.

**LC-MS untargeted metabolomics determination and analysis**

The instrument platform for LC-MS analysis was the ultra-high- performance liquid chromatography-triple time-of-flight mass spectrometry

(UPLC-Triple-TOF) system from company AB SCIEX LLC.

Chromatographic analysis was performed on a Waters Acquity Ultra highPerformance LC system equipped with a BEH C18 column (2.1 × 100 mm, 1.7 μm, Waters Corp., Milford, USA). The autosampler and column compartment were maintained at 4 °C and 40 °C, respectively. The mobile phase consisted of solvents A(H_2_O + 0.1% formic acid) and B (acetonitrile/Isopropanol 1:1 + 0.1% formic acid). The elution gradient program for fecal samples was: 5–20% B for 3 min; 20–95% B from 3 to 9 min; keeps 95% B from 9 to 13 min; B decreases from 95% to 5% for 0.1min; keeps 5% B from 13.1 to 16 min. the flow rate was 0.40 mL/min and column compartment were maintained at 40 °C .

Mass spectrawere acquired on positive and negative ion scanning mode; mass scanning range(m/z): 50-1000. Ion spray voltage: positive ion voltage: 5000V; negative ion voltage: 4000V; de-cluster voltage: 80V; spray gas: 50psi; auxiliary hot gas: 50psi; curtain gas: 30psi; ion source heating temperature: 500℃; 20-60V cycle collision energy.

Chromatographic condition: metabolite profiling was conducted by Metabolon Inc (Durham, NC) as previously described. All methods utilized Waters ACQUITY ultra-performance liquid chromatography (UPLC) and a Thermo Scientific Q-Exactive high resolution/accurate MS interfaced with a heated electrospray ionization source and Orbitrap mass analyzer operated at 35,000 mass resolution.

LC-MS data were imported into metabolomics processing software Progenesis QI (Waters Corporation, Milford, USA) for baseline filtering, peak recognition, integration, retention time correction, and peak alignment. The mass-to-charge ratio, peak intensity data matrix, and data matrix were used to remove the missing values according to the 80% rule. The response intensity of sample spectral peak was normalized by summation normalization method, and the obtained normalized data matrix was processed by log10 logarithm. The MS mass spectrum information that is the final data matrix matched the public databases and metabolic HMDB (http://www.hmdb.ca/) and Metlin (https://metlin.scripps.edu/). Finally, the metabolite information was obtained.

The data were analyzed on the online platform of Majorbio Cloud Platform (www.majorbio.com). Principal component analysis (PCA) was used to identify the most important elements and reveal cluster separation between the different groups. Partial Least Squares Discrimination Analysis（PLS-DA analysis）is a supervised analysis method, similar to PCA in principle, but samples must be specified and grouped during the analysis. In addition, Student’s t-test was performed. The significantly different metabolites were selected based on the variable importance in projection(VIP) and P values of the Student’s t-test. VIP > 1 and P < 0.05 were considered statistically different. The KEGG database (https://www.kegg.jp/kegg/pathway.html) analyzed the differences in metabolites involved in pathways between the groups. KEGG enrichment analysis was performed through the Python software package scipy.stats (https://docs.scipy.org/doc/scipy/), and the biological pathway was obtained through Fisher’s exact test, which uses topology analysis to ascertain the pathways that are significantly altered between the two class models. Statistical analyses were performed using R software package ropls (Version1.6.2).
