# Supplementary material for: Intra-tumoral microbial community profiling and associated metabolites alterations of TNBC
Source: Front Oncol. 2023 Oct 12;13:1143163. doi: 10.3389/fonc.2023.1143163 (PMC10602718; doi:10.3389/fonc.2023.1143163)
Supplement: Supplementary file 2 [file Table_1.docx]

**Supplementary Table 1 Clinical information of relationship between TNBC and non-TNBC**

| Variables | TNBC | Non-TNBC | *p* |
| --- | --- | --- | --- |
| Age |  |  |  |
| ＜50 | 8 | 4 | 0.429 |
| ≥50 | 5 | 5 |  |
| Size |  |  |  |
| ＜2cm | 7 | 6 | 0.548 |
| ≥2cm | 6 | 3 |  |
| BMI |  |  |  |
| ＜24Kg/m^2^ | 4 | 4 | 0.512 |
| ≥24Kg/m^2^ | 9 | 5 |  |
| Parity |  |  |  |
| ＜2 | 10 | 7 | 0.962 |
| ≥2 | 3 | 2 |  |

*Statistically significant at P < 0.05.
