# Supplementary material for: Intra-tumoral microbial community profiling and associated metabolites alterations of TNBC
Source: Front Oncol. 2023 Oct 12;13:1143163. doi: 10.3389/fonc.2023.1143163 (PMC10602718; doi:10.3389/fonc.2023.1143163)
Supplement: Supplementary file 3 [file Table_2.docx]

**Supplementary Table 2 Clinical pathological analysis of relationship between TNBC and non-TNBC**

| Variables | TNBC | Non-TNBC | Χ^2^ | *p* |
| --- | --- | --- | --- | --- |
| Age |  |  |  |  |
| ＜50 | 35(51.5) | 33(48.5) | 3.922 | 0.048* |
| ≥50 | 23(41.8) | 32(58.2) |  |  |
| Size |  |  |  |  |
| ＜3 | 28(57.1) | 21(42.9) | 5.413 | 0.020* |
| ≥3 | 30(40.5) | 44(59.5) |  |  |
| TNM |  |  |  |  |
| Ⅰ/Ⅱ | 42(48.3) | 45(51.7) | 0.322 | 0.570 |
| Ⅲ-Ⅳ | 16(44.4) | 20(55.6) |  |  |
| Grade |  |  |  |  |
| Ⅰ/Ⅱ | 42(40.4) | 62(59.6) | 41.087 | 0.001* |
| Ⅲ | 16(84.2) | 3(15.8) |  |  |
| Lymphatic  metastasis |  |  |  |  |
| No | 35(51.5) | 33(48.5) | 1.816 | 0.178 |
| Yes | 23(41.8) | 32(58.2) |  |  |
| TILs |  |  |  |  |
| Negative  (＜10%) | 49(50) | 49(50) | 3.998 | 0.046* |
| Positive  (≥10%) | 9(36) | 16(64) |  |  |
| PDL1 |  |  |  |  |
| Negative | 34(45.3) | 41(54.7) | 0.501 | 0.479 |
| Positive | 24(50) | 24(50) |  |  |

*Statistically significant at P < 0.05.
