# Supplementary figures and images for: Intra-tumoral microbial community profiling and associated metabolites alterations of TNBC
Source: Front Oncol. 2023 Oct 12;13:1143163. doi: 10.3389/fonc.2023.1143163 (PMC10602718; doi:10.3389/fonc.2023.1143163)

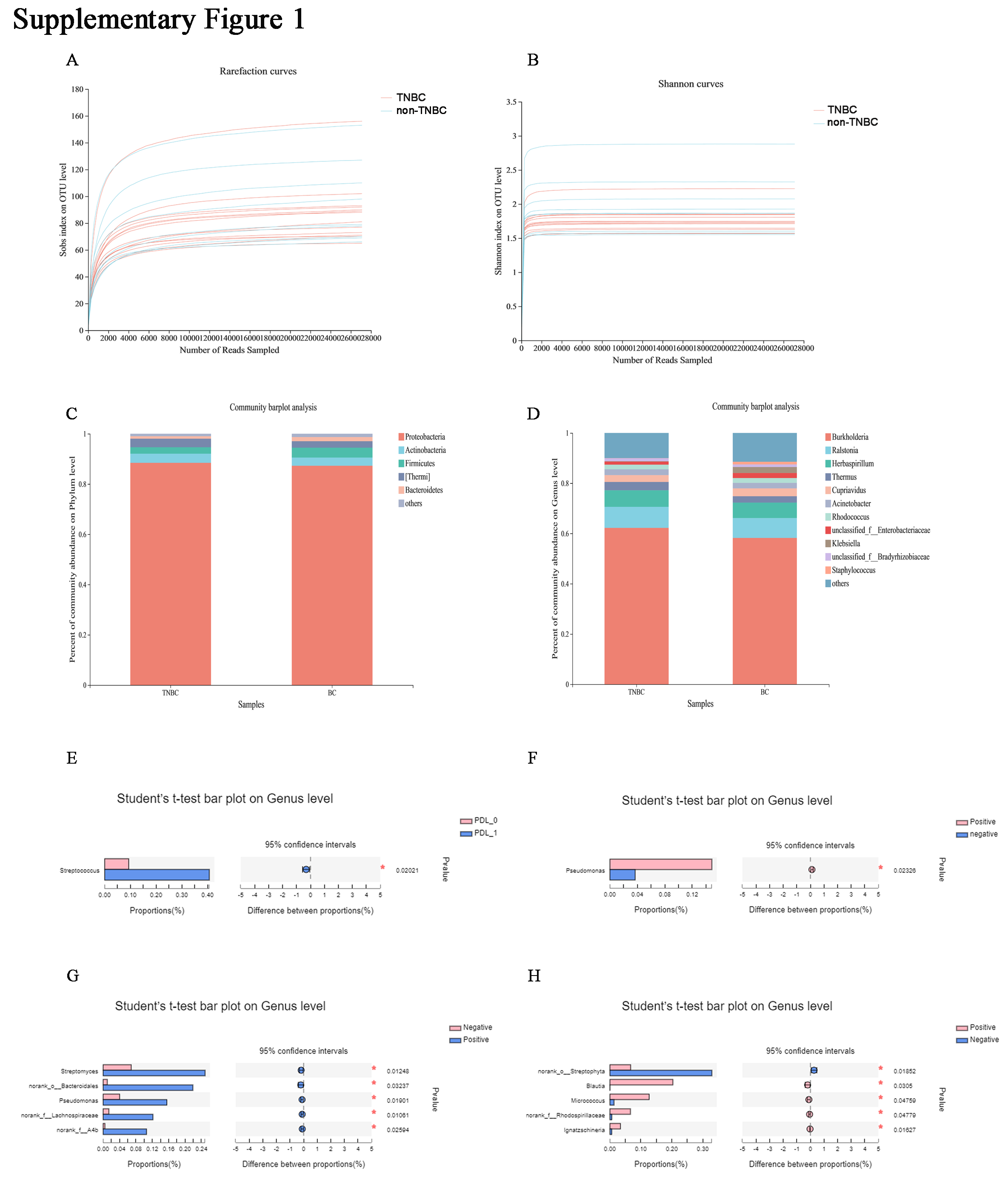

Supplement: Supplementary Figure 1 — Composition and differences of tumor microbiota in different clinical indices. [file Image_1.tif]

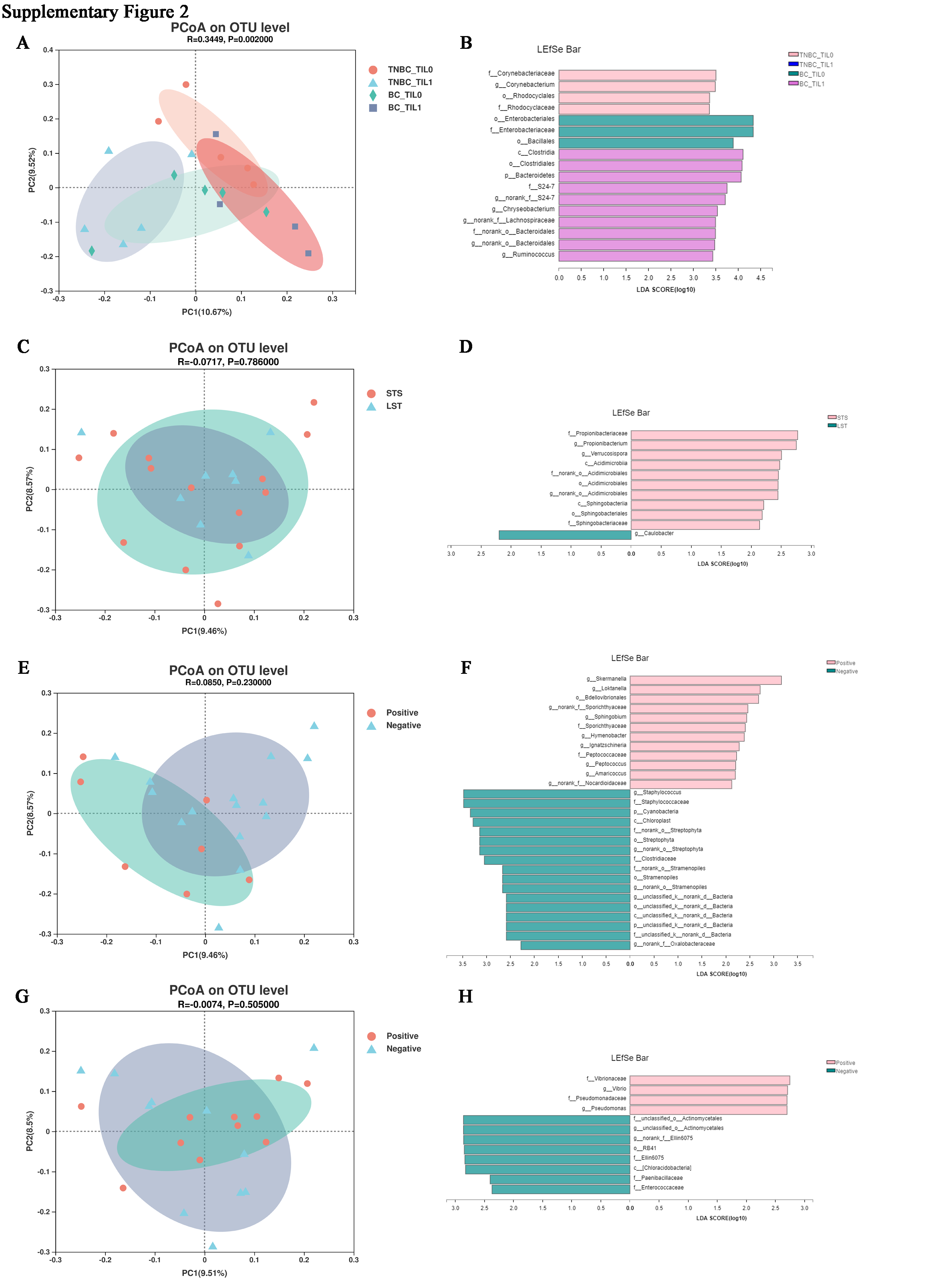

Supplement: Supplementary Figure 2 — The diversity and abundance of microbiota in other related elements. [file Image_2.tif]

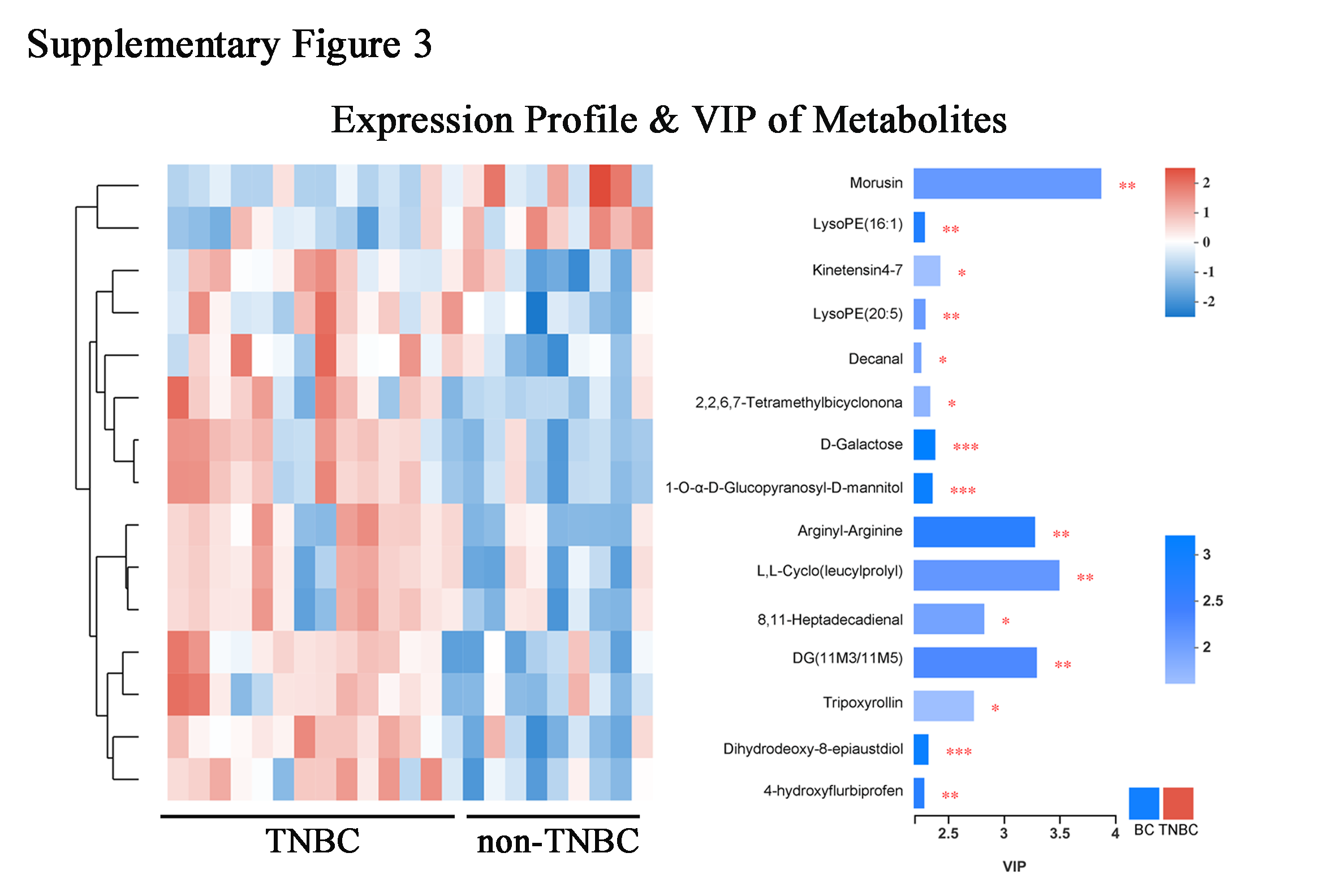

Supplement: Supplementary Figure 3 — Variable importance in projection (VIP) scores of tumor metabolites in TNBC and non-TNBC groups. [file Image_3.tif]

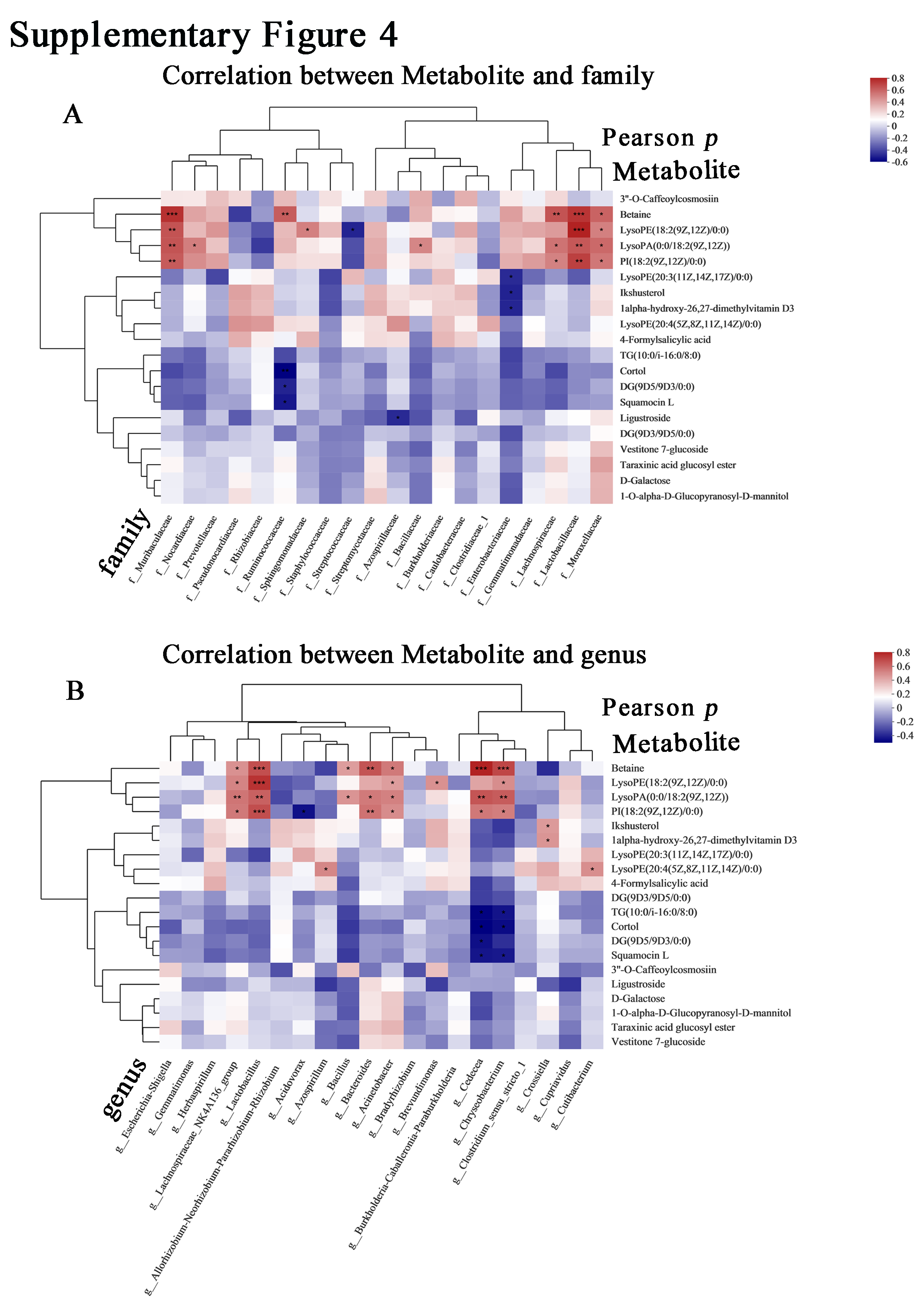

Supplement: Supplementary Figure 4 — Correlation analysis of microbes and metabolites. [file Image_4.tif]

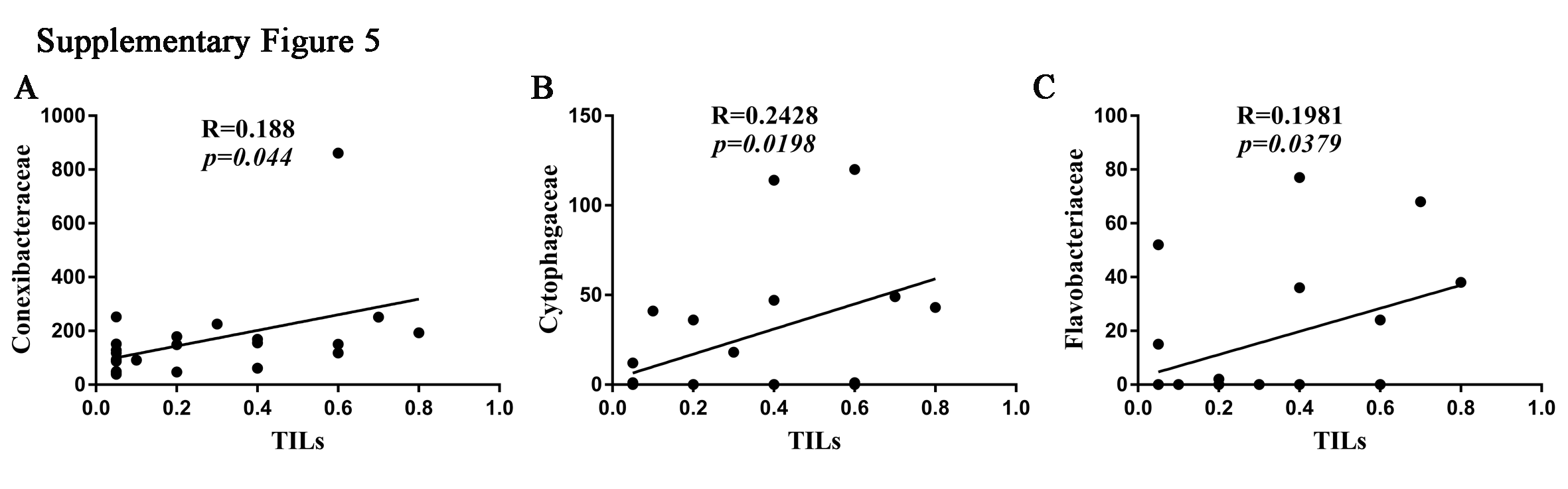

Supplement: Supplementary Figure 5 — The correlation between the intra-tumoral microbiota and clinicopathological characteristics. [file Image_5.tif]
